# Pronounced strain-specific chemosensory receptor gene expression in the mouse vomeronasal organ

**DOI:** 10.1186/s12864-017-4364-4

**Published:** 2017-12-12

**Authors:** Kyle Duyck, Vasha DuTell, Limei Ma, Ariel Paulson, C. Ron Yu

**Affiliations:** 1 Stowers Institute for Medical Research, 1000 East 50th Street, Kansas City, MO 64110 USA; 2 Department of Anatomy and Cell Biology, University of Kansas Medical Center, 3901 Rainbow Boulevard, Kansas City, KS 66160 USA; 3 Redwood Center for Theoretical Neuroscience, University of California, 567 Evans Hall, Berkeley, 94720 USA

**Keywords:** Vomeronasal, Pheromone, Sexual dimorphic, Strain, Innate behavior, G-protein coupled receptor, Transcriptome

## Abstract

**Background:**

The chemosensory system plays an important role in orchestrating sexual behaviors in mammals. Pheromones trigger sexually dimorphic behaviors and different mouse strains exhibit differential responses to pheromone stimuli. It has been speculated that differential gene expression in the sensory organs that detect pheromones may underlie sexually-dimorphic and strain-specific responses to pheromone cues.

**Results:**

We have performed transcriptome analyses of the mouse vomeronasal organ, a sensory organ recognizing pheromones and interspecies cues. We find little evidence of sexual dimorphism in gene expression except for *Xist,* an essential gene for X-linked gene inactivation. Variations in gene expression are found mainly among strains, with genes from immune response and chemosensory receptor classes dominating the list. Differentially expressed genes are concentrated in genomic hotspots enriched in these families of genes. Some chemosensory receptors show exclusive patterns of expression in different strains. We find high levels of single nucleotide polymorphism in chemosensory receptor pseudogenes, some of which lead to functionalized receptors. Moreover, we identify a number of differentially expressed long noncoding RNA species showing strong correlation or anti-correlation with chemoreceptor genes.

**Conclusions:**

Our analyses provide little evidence supporting sexually dimorphic gene expression in the vomeronasal organ that may underlie dimorphic pheromone responses. In contrast, we find pronounced variations in the expression of immune response related genes, vomeronasal and G-protein coupled receptor genes among different mouse strains. These findings raised the possibility that diverse strains of mouse perceive pheromone cues differently and behavioral difference among strains in response to pheromone may first arise from differential detection of pheromones. On the other hand, sexually dimorphic responses to pheromones more likely originate from dimorphic neural circuits in the brain than from differential detection. Moreover, noncoding RNA may offer a potential regulatory mechanism controlling the differential expression patterns.

**Electronic supplementary material:**

The online version of this article (10.1186/s12864-017-4364-4) contains supplementary material, which is available to authorized users.

## Background

In terrestrial animals, pheromones and olfactory cues mediate some key social behaviors [1–3]. Pheromones carry information about sex, reproductive status, genetic background, and individuality of the animals [1, 4]. In many vertebrate species, the vomeronasal organ (VNO) has evolved to specialize in detecting pheromone cues [5, 6]. The recent finding that the VNO responds to cues from other species expands its role in chemosensory perception [7]. In mice, the VNO expresses three major families of G protein coupled receptors: V1rs, V2rs, and formyl peptide receptors (FPRs) [8–13]. Additionally, some odorant and taste receptors are also detected in the VNO.

It has long been recognized that sexually dimorphic behaviors in male and female mice can be triggered by pheromone cues. For example, urine from mature female mice elicits sexual arousal in males, but suppresses sexual maturation and delays estrus cycle in females [14]. The origin of these sexually dimorphic behaviors may arise from brain circuitry that processes pheromone information, the differential recognition of pheromone signals by the sensory organs, or both. Previous studies have found moderate differences between male and female animals in the expression of a few genes in the VNO [15]. However, these studies have examined a single strain of mice, which may not be generalized to mice of different genetic backgrounds. True sexual dimorphism should be detected across different strains.

The patterns of activity in the mouse VNO can encode information about sex, genetic background and individuality of the carrier [16], as well as other species [7]. Several observations suggest that VNO is central in orchestrating innate behaviors. For example, some strains of mice exhibit the Bruce effect, when the presence of a stud male from a different strain causes a newly mated female to abort pregnancy [17]. Exhibition of the Bruce effect depends on not only the recognition of sex, but also strain information, by the VNO [18, 19]. Animals also display kinship recognition and respond stereotypically to cues from animals of different genetic backgrounds. Mice prefer sexual partners of a different genetic background [20, 21]. It is unknown whether kinship recognition and mating preferences directly arise from differential recognition of chemosensory cues mediated at the level of sensory organ.

The vomeronasal receptors are among the fastest evolving genes [22–35]. Comparison of receptor diversity among different species demonstrates highly divergent family members and receptor sequences [26, 28, 31–33, 36]. The diversity of receptor likely accommodates the variety of pheromone molecules. It is possible that co-evolution of pheromones and their receptors results in differential behavioral responses in various strains to influence mate choice, mating frequency and other reproductive behaviors. Differential expression of the receptors and associated proteins may also have a direct impact on how pheromones are recognized. In this study, we analyze VNO transcriptomes of both sexes from four inbred mouse strains. These analyses reveal a rich array of genes that are differentially expressed by the VNO with implications on how pheromones cues can be differentially recognized by different strains of mice.

## Results

### Lack of significant sexual dimorphism in VNO gene expression

We dissected VNO neuroepithelia from 6-week-old male and female animals of C57BL/6 (B6), 129Sv/J (129), SJL and SWR strains. The widely used B6 and 129 strains are derived from the Lathrop and Castle lineages, respectively [37]. In comparison, SJL and SWR lines descend from the Swiss lineage and are closely related to each other. We reason that sampling from these four strains may provide information about strain and sex difference in VNO gene expression.

**Fig. 1:**
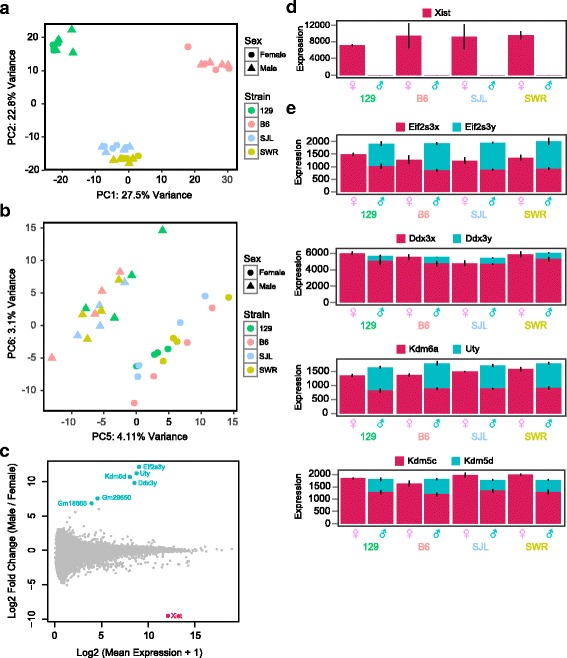
Sex linked gene expression in the VNO. **a** and **b** Principal components analysis (PCA) of all expressed genes in VNO of B6, 129, SWR and SJL strains. **c** MA plot of gene expression in VNOs from male and female mice. Y-axis indicates the maximal value of fold change (FC) between male and female in log2 scale. Genes that exhibit significant DE are highlighted in color (weighted FC > 2; *p* < 0.01). **d** Bar plot of mean normalized expression of *Xist* in male and female mice. **e** Stacked bar plot of mean normalized value of Y chromosome linked genes and their X chromosome homologs. Genes expressed from the X and Y chromosomes are labeled red and teal, respectively. Error bars represent standard deviation of expression values

We extracted total RNA from individual VNO neuroepithelia and performed ribo-depletion to remove ribosomal RNA from the samples prior to library construction. Routine RNAseq was performed on HiSeq platform and high-quality reads were mapped to GRCm38 (mm10) mouse reference genome (Additional file 1: Figure S1). In total, we identified 44,957 genes as expressed by any of the samples. Principal components analysis (PCA) of the dataset indicated that the samples were well separated according to strains (Fig. 1a), with principal component 1 (PC1, 27.5% variance) separating B6 and 129 from each other and the Swiss strains, and PC2 (22.8% variance) separating 129 from both the B6 and Swiss strains. Within each strain, however, male and female samples were intermingled (Fig. 1a). Analyses of the first four PCs, which accounted for 72.9% of the variance, did not reveal an axis that separated the sexes. Only for PC5 and PC6 (4.11% and 3.1% variances, respectively) did we observe clear separation by sex for all samples (Fig. 1b). This result indicated that sex did not contribute significantly to the variance of gene expression in VNO, although some of the genes indeed showed sexual dimorphic expression.

Previous studies discovered limited sexual dimorphism in gene expression from the olfactory tissues of the B6 strain [15]. However, it was not clear whether the observed sexual dimorphism was also present in other strains as well. We reasoned that for a gene to be considered truly sexual dimorphic, the differential expression between male and female should be consistently observed across all strains. By comparing the male and female samples from all four strains, we found seven genes emerge as differentially expressed (DE) between the sexes with fold change (FC) greater than 2, or Log_2_ fold change (LFC) greater than 1 (*p* < 0.01) (Fig. 1c). Among these were *Xist* (Fig. 1d), an X-linked non-coding RNA gene that plays an essential role in X-inactivation [38], and six Y chromosome genes: *Gm18665, Gm29650, Eif2s3y*, *Ddx3y*, *Kdm5d*, and *Uty* (*Kdm6c*). When we examined these Y chromosome genes, we found that the expression levels of their X allele homologs were slightly lower in males than females (FC < 2; Fig. 1e). Moreover, apart from *Eif2s3*, the expression of the Y chromosome counterpart of the genes in males largely compensated the differences between male and female samples (Fig. 1e). These results suggested that the X-allele genes did not escape dosage-compensation in female VNO. After taking into account the expression of their Y chromosome counterparts, the functions of these genes were not sexually dimorphic. We found no other transcripts, including those related to chemosensory perception such as odorant receptor, vomeronasal receptor or pheromone binding protein genes, to be differentially expressed in the VNO between the sexes. Thus, *Xist* was the only gene exhibiting sexually dimorphic expression in the VNO.

### Differential gene expression among strains

**Fig. 2:**
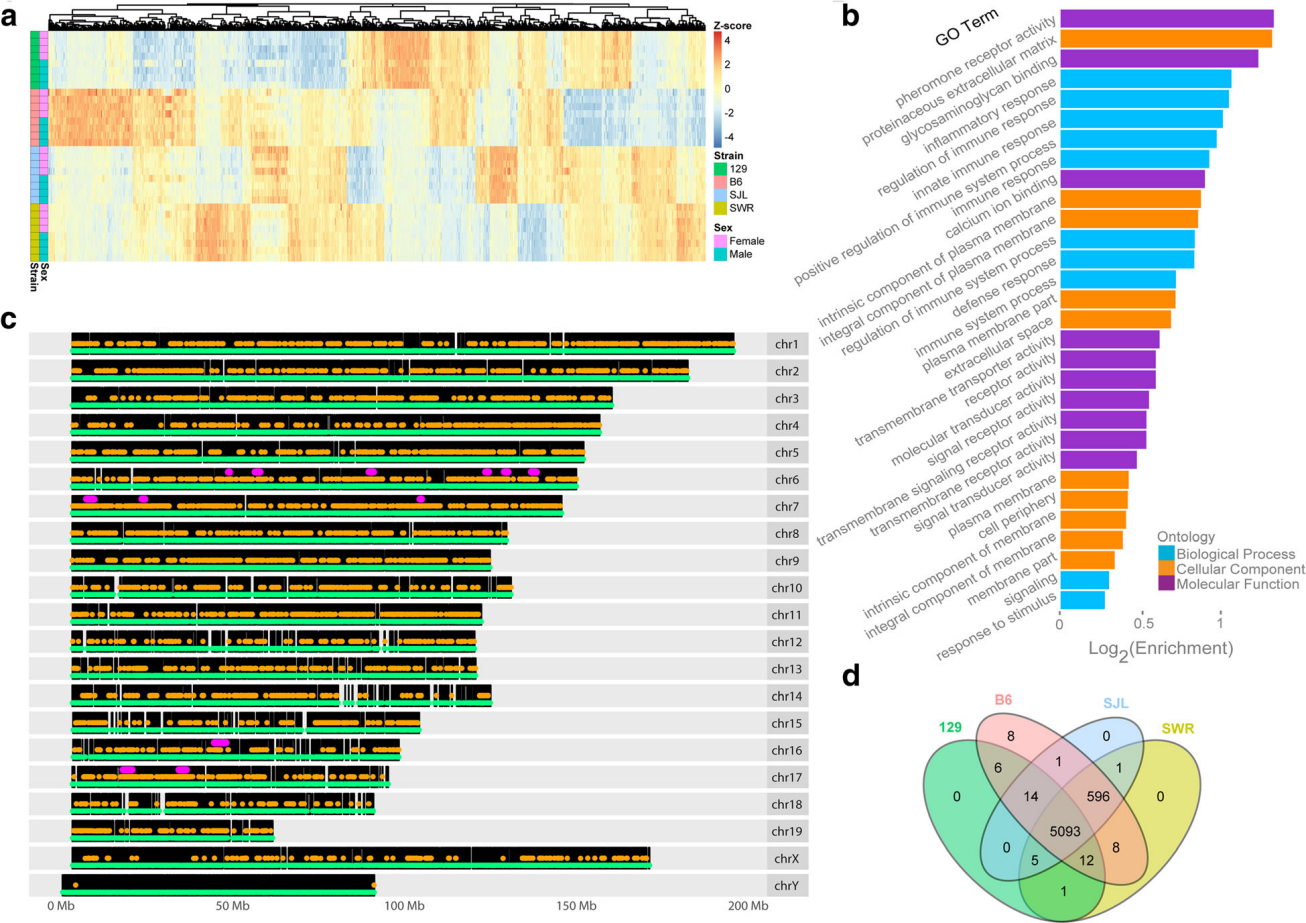
Differential expression of genes among strains. **a** Heatmap of top 1000 differentially expressed (DE) genes in four strains. DE genes are ranked by q-value. **b** GO terms analysis of DE genes. **c** Genomic locations of DE genes on all chromosomes. Each black vertical line indicates an annotated gene. Green and yellow dots indicate expressed and differentially expressed genes, respectively. Purple ellipses on chromosomes 6, 7, 16, and 17 highlight ‘hot spots’ regions that are enriched for DE genes. **d** Venn diagram showing the number of genes specifically expressed by a single strain, or shared by different strains

We next examined whether gene expression in VNO was different among strains. Of the 44,957 genes expressed in the VNO, we identified a list of 5745 genes (12.8% of all expressed) that were DE among the strains with FC > 2, and false discovery rate (FDR) <0.05 (Fig. 2a). Of these DE genes, 1644 were annotated as either gene models (Gm) or Riken (Rik) genes. These putative genes constituted the largest subgroup (28.6%) of DE genes with no known function. It was yet to be determined how these transcripts affected VNO functions.

For the remaining 4101 DE genes that had functional annotations, we performed a gene ontology (GO) analysis to investigate a possible enrichment of the GO terms in certain categories (Fig. 2b). This analysis indicated that G-protein coupled receptor (GPCR) activity and immune system related genes dominated the list. Enriched GO terms of the Biological Process category were related to the regulation of immune, stimulus, and inflammatory responses, as well as signaling (classic Fisher, *p* < 1e-23). In the Molecular Function category, GO terms were highly enriched for binding of calcium and glycosaminoglycan, activity of pheromone, transmembrane, and signal receptors, and transmembrane transporter activity (classic Fisher, p < 1e-12). GO terms in the Cellular Component category were enriched for the cellular periphery, plasma membrane, and extracellular space (classic Fisher, p < 1e-25).

Whereas differentially expressed genes were located throughout the entire genome, some chromosomal regions appeared to contain high numbers of DE genes. By applying a sliding window across all the expressed genes on each chromosome, we identified 12 “hot spots” -- genomic regions in which there were a larger percentage of DE genes than random scattering would predict (Poisson test, FDR < 0.05). Interestingly, these clusters are enriched in genes from the chemoreceptor and immune system related gene families. We identified six hot spots on Chr. 6, three on Chr. 7, one on Chr. 16, and two on Chr. 17 (Fig. 2c, Table 1). Three of the six Chr. 6 hotspots, and two of the three Chr. 7 hotspots contained vomeronasal receptors, including vmn1r (Chr. 6) and vmn2r (Chr. 7). Of the 2 hotspots on Chr. 17, the largest one corresponded to a locus enriched in vmn2r genes.

**Table 1:** Hot Spot of Differentially Expressed Genes

Chr	Start	End	Span (MB)	Expr. Genes	DE genes	% DE	Prominent Gene Families
6	48,448,229	48,754,210	0.31	25	11	44%	GIMAP
6	56,172,928	57,664,632	1.49	39	14	35.9%	Vmn1r (Clade C)
6	89,316,314	90,600,203	1.28	46	19	41.3%	Vmn1r (Clades A, B)
6	123,195,632	124,082,601	0.89	27	11	40.7%	Clec, Vmn2r (Clade B)
6	128,648,576	129,740,484	1.09	62	28	45.2%	Clec, Klr
6	136,506,167	138,079,916	1.57	30	12	40%	NA
7	7,171,330	9,389,264	2.22	67	32	47.8%	Vmn2r (Clade A4)
7	23,272,801	24,143,241	0.87	41	18	43.9	Vmn1r (Clade D)
7	104,140,623	104,601,779	0.46	32	13	40.6	Olfr, Trim
16	44,347,121	47,758,671	3.41	40	17	42.5	Cd200, Cd200r
17	17,830,352	20,405,756	2.58	44	19	43.2	Fpr, Vmn2r (Clade A8)
17	34,031,812	36,198,513	2.17	122	47	38.5	Btnl, MHC (Clades IIa, IIb, Ia, Ib)
Hot Spot Totals			18.34	575	241	41.9%	
Whole Genome				44,957	5745	12.8%	

The remaining hotspots largely contained immune system related genes. Three hotspots on Chr. 6 contained genes from the GIMAP, Clec, Klr families of genes. The hotspots on Chr. 7 and Chr. 16 contained Trim, and CD200/CD200 receptor genes, respectively. On Chr. 17, a 2.17 Mb hot spot was enriched with Butyrophilin-like and MHC class 1b, 2a, and 2b genes, with 20 of 28 of the MHC genes differentially expressed. The downstream end of the hotspot was enriched for MHC class 1b genes. This region was the most densely packed, with 35 expressed genes in a region less than 1 Mb in length, with almost half of them differentially expressed. In total, the hot spots covered 18.34 Mb and 575 expressed genes, 241 (41.9%) of which were DE. This percentage contrasts to the whole genome with an average of 12.8% DE genes.

Some differentially expressed genes were present in all strains but at different levels. Others were expressed exclusively in some strains but not others. 5093 (89%) of the DE genes were expressed by all four strains (Fig. 2d). The remaining 11% had no expression in the VNO of both sexes in at least one strain. Of these, eight genes were expressed solely in C57BL/6, and 627 genes were excluded in one strain.

### Chemosensory receptor expressions in different strains

**Fig. 3:**
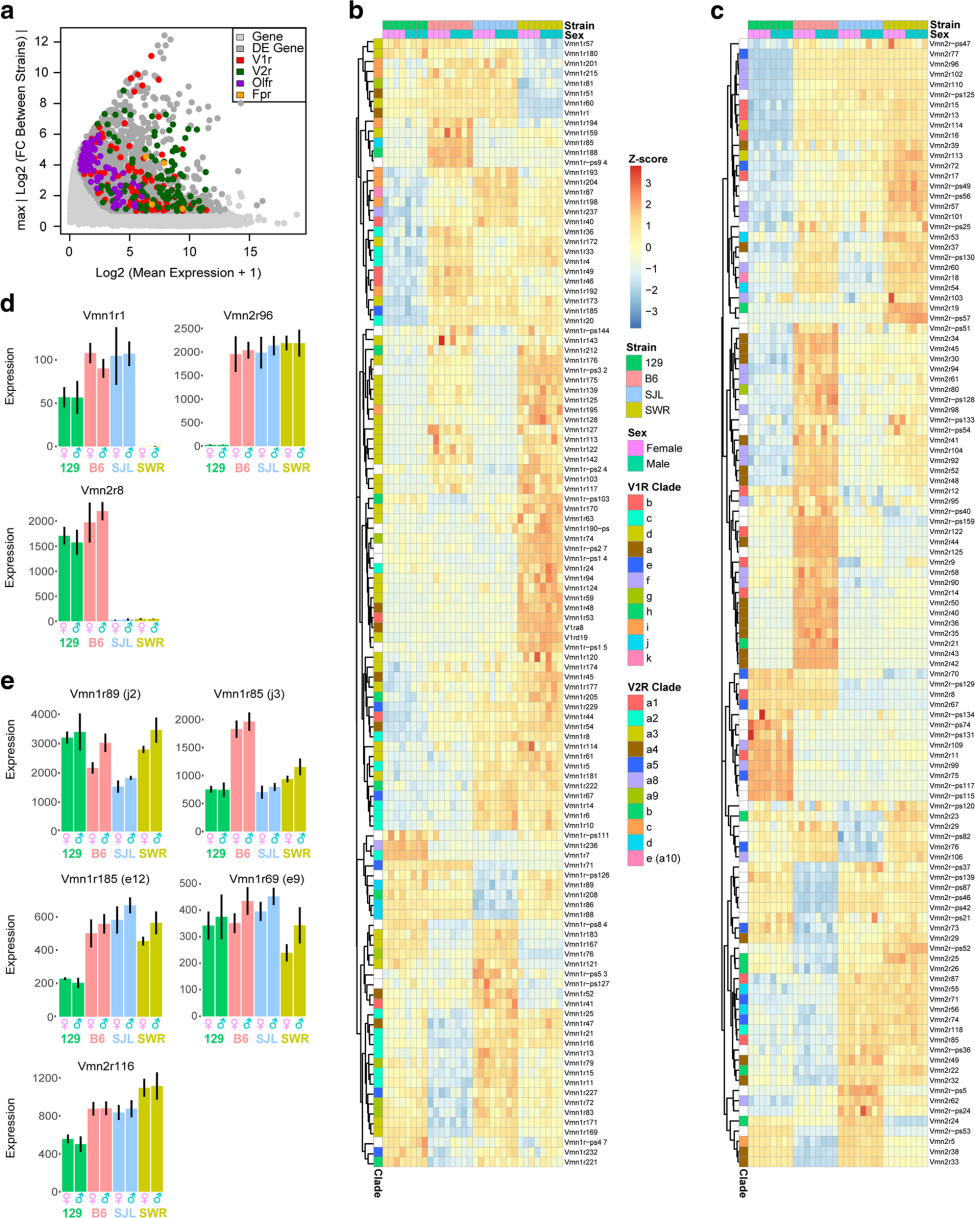
Differential expression of chemosensory receptor genes. **a** MA plot highlighting DE chemosensory receptor genes. Y-axis indicates the maximal value of FC between any two strains in log2 scale. Chemosensory receptors genes are highlighted, including 114 V1r (red), 111 V2r (green), 141 Olfr (purple), and 4 Fpr (orange). **b-c** Heatmaps showing the DE chemoreceptor genes, including V1rs (B) and V2rs (C). Each clade is color-coded. **d** Example bar plots showing expression profiles for highly DE V1rs & V2rs across different strains. **e** Bar plots showing the expression levels of receptors identified as sex pheromone detecting receptors. Error bars represent standard deviation of expression values

In our analyses, GPCRs (453 out of 5745) constituted a large group of DE genes (Fig. 3a), which included 114 V1r (Fig. 3b), 111 V2r (Fig. 3c), 141 olfactory receptors (Additional file 2: Figure S2), 4 formyl peptide receptors, and 2 taste receptor genes. Differentially expressed V1r genes were found in all clades (A - K) except L, which contains only one gene *Vmn1r70* (Fig. 3b). The DE V2r genes were also found in all clades (A1- A5, A8, A9, B, C, D, and E) except for clade A6, which also contains only one gene, *Vmn2r120* (Fig. 3c).

Interestingly, we observed a completely lack of expression of some chemosensory genes in one or more strains (Fig. 3d and Additional file 3:Figure S3). Some genes were expressed in a mutually exclusive fashion among the tested strains (Additional file 3: Figure S3). In the V1r family, for example, *Vmn1r188* was expressed exclusively in B6, while *Vmn1r76* was expressed in all strains except SWR. In the V2r family, *Vmn2r-ps24* was expressed in all but the 129 strain. We also observed a similar scenario in the DE olfactory receptor genes. *Olfr279* and *Olfr116* were expressed in all but 129 mice. Overall, among the DE chemoreceptor genes, 12.3% (14/114) of the V1rs, 8.1% (9/111) of the V2rs, and 65.2% (92/141) of the ORs completely lacked expression in at least one strain. Some of the differentially expressed VRs show single nucleotide polymorphisms (SNPs) with both synonymous and non-synonymous changes. (Additional file 4: Figure S4).

The expression level of different VR genes varied widely. Some clades, such as V1r clade J, E and F, were expressed at higher levels than others (Fig. 3 and Additional file 5: Figure S5). Clade E and J members were shown to recognize female specific cues that identify the sex and the reproductive status of female mice (Fig. 3e) [39]. The function of the V1rf genes remained unknown.

FPRs are a family of chemosensory receptors expressed in the VNO implicated in the recognition of the health statuses of the animals [8, 9, 40]. *Fpr-rs3* had the strongest expression among all FPR genes, which was about 3-fold higher than other FPR. It was also one of four FPR genes differentially expressed. In addition to differential expression, we also found SNPs in FPR genes specific to 129 strain mice (Additional file 6: Figure S6). SNPs in the coding regions of *Fpr-rs3, Fpr-rs4* and *Fpr-rs6* altered protein sequences. One synonymous SNP was found within the protein-coding region of *Fpr3*. The changes in both expression levels and coding sequences implied that the recognition of FRP ligands were likely to be different between 129 and the other strains.

Of the 141 olfactory receptors, only a few data points have more than 1 transcripts per million, indicating that their expression is either limited to an extremely small population of cells or is from leakage. Besides the classical chemosensory receptors, we identified 409 genes that were expressed in the VNO of at least one strain, and had GO terms related to GPCR activity or one of its children terms. Of the 409 expressed genes in this group, 138 were differentially expressed between the strains, however none was shown directly to be involved in VNO signaling (data not shown).

### VRs detecting sex pheromones

Only a handful of VRs have been assigned functions in pheromone signaling. This made it difficult to assess whether the differentially expressed receptors could affect pheromone-dependent behaviors. Previous studies have identified several receptors involved in sexually dimorphic behavior in mice [39, 41, 42]. We, therefore, specifically examined *Vmn1r69* (*V1re9*) and *Vmn1r185* (*V1re12*), two receptors known to respond to female sex-specific pheromone cues; *Vmn1r85* (*V1rj3*) and *Vmn1r89* (*V1rj2*), two receptors known to recognize estrus cues; and *Vmn2r116* (*V2rp5*), a receptor for the male-specific ESP-1 peptide (Fig. 3e) [39, 41, 42]. We found all four V1r genes in all strains suggesting the critical roles of these receptors in mating behavior. Three of these genes, *Vmn1r185* and *Vmn1r85*, *Vmn1r89*, were differentially expressed among the strains, with *Vmn1r185* expressed significantly less in the VNO of 129 strain mice, and *Vmn1r85* expressed at higher level in B6 mice than any other strains. Expression of *Vmn1r89* was slightly higher in male VNO of all strains, but the difference was not statistically significant. No genes exhibited preferential expression in the females.

We observe high levels of polymorphism in Vmn2r116 for 129 strain mice, although the difference in expression between strains is not significant given our stringent threshold of FC > 2 (Fig. 3e). There were six SNPs within the reading frame, five of which resulted in non-synonymous amino acid changes, including a Gly to Asp substitution within the predicted 7-TM domain. In contrast, no SNPs within the reading frames of *Vmn1r185* or *Vmn1r89* were detected. *Vmn1r69* contained only two SNPs, both found only in the Swiss mice, and only one of which resulted in a change in amino acid sequence. *Vmn1r85* contained no synonymous polymorphisms within the ORF.

### Functionalized Pseudogenes

**Fig. 4:**
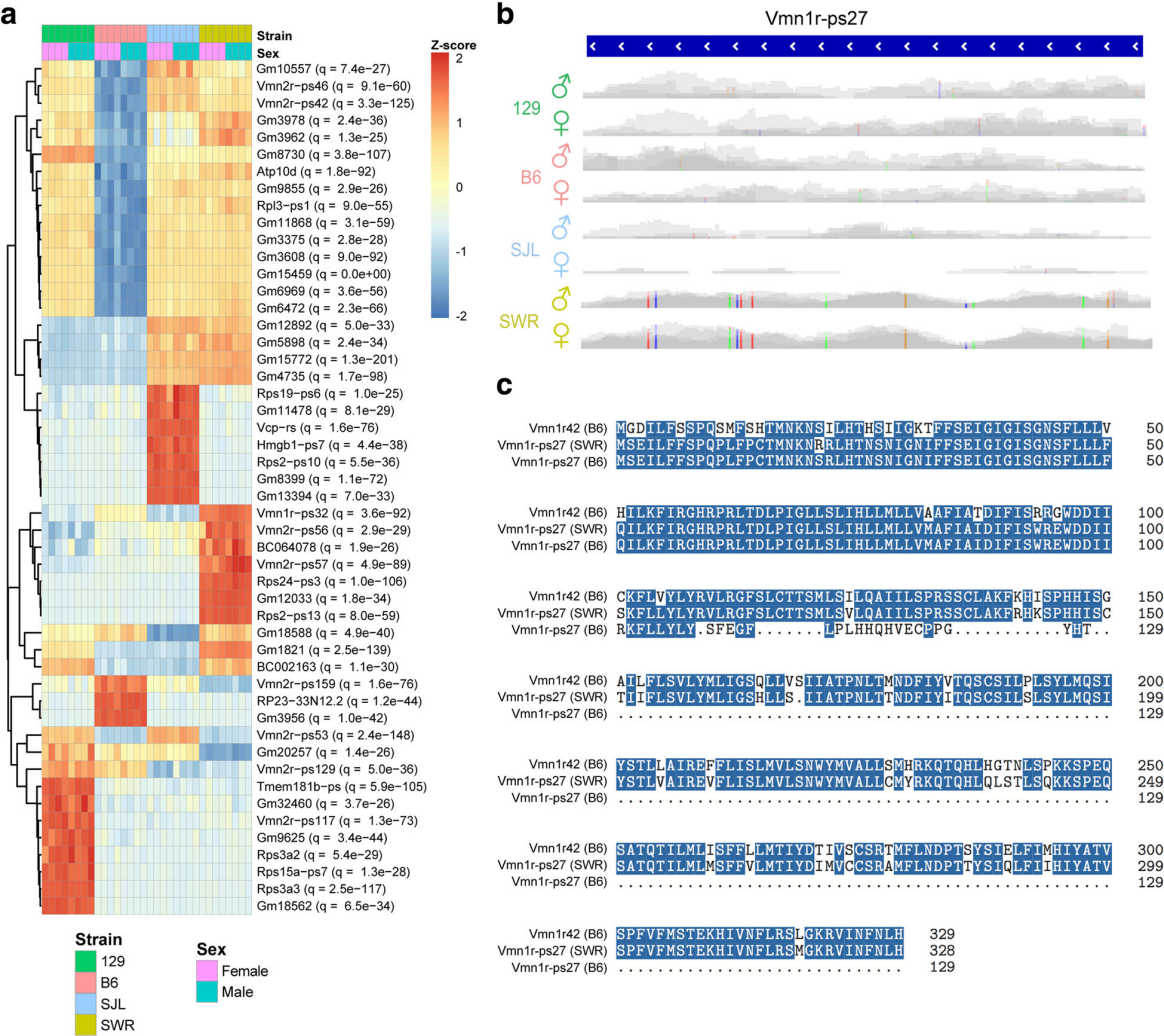
Differential expression of pseudogenes and gene model transcripts. **a** Heatmap of expression profiles of top 50 pseudogenes and gene models that are DE across strains. DE genes are ranked by q-value. **b** Track view of Vmn1r-ps27. The expression levels are of the same scale and 10 SNVs (color bars) are indicated with base substitutions represented as follows: thymine as red, guanine as brown, cytosine as blue, and adenine as green. **c** Translated sequence of SWR Vmn1r-ps27 indicates that it is a full-length V1r with 84% identity to Vmn1r42

We identified a list of 504 DE genes that were annotated as pseudogenes in the reference genome. B6 had the lowest pseudogene expression (Fig. 4a). Many of these pseudogenes contained SNPs, some of which led them to encode functional proteins. Two Vmn1r pseudogenes, *Vmn1r-ps27*, and *Vmn1r-ps32*, as well as one Vmn2r pseudogene, *Vmn2r-ps53*, encoded functional receptors because of insertions that changed the reading frame and/or SNPs that removed stop codons. *Vmn1r-ps27* was expressed over 2-fold higher in SWR than in any other strain. It contained ten SNPs solely found in the SWR strain (both male and female samples) (Fig. 4b-c, Additional file 7: Figure S7). These SNPs resulted in an ORF over the entire gene length to encode a 329- amino acid protein that shared 84% protein identity (91% nucleic acid identity) with Vmn1r42 (Fig. 4c and Additional file 7: Figure S7). *Vmn1r-ps32*, which was expressed over 3-fold higher in SWR than in any other strain, contained a C insert 359 bp from the start codon that restored the reading frame such that the ORF encoded a 318-amino acid protein with 95% protein identity and 97% nucleic acid identity to *Vmn1r45*. We suspect that this phenomenon is more widespread than these two examples. However, due to the lack of complete reference genome for 129, SWR and SJL at the time of the study, we are not able to test whether all B6 psuedogenes listed in Fig. 4a have functional counterparts in the other three strains.

### Immune system related genes

**Fig. 5:**
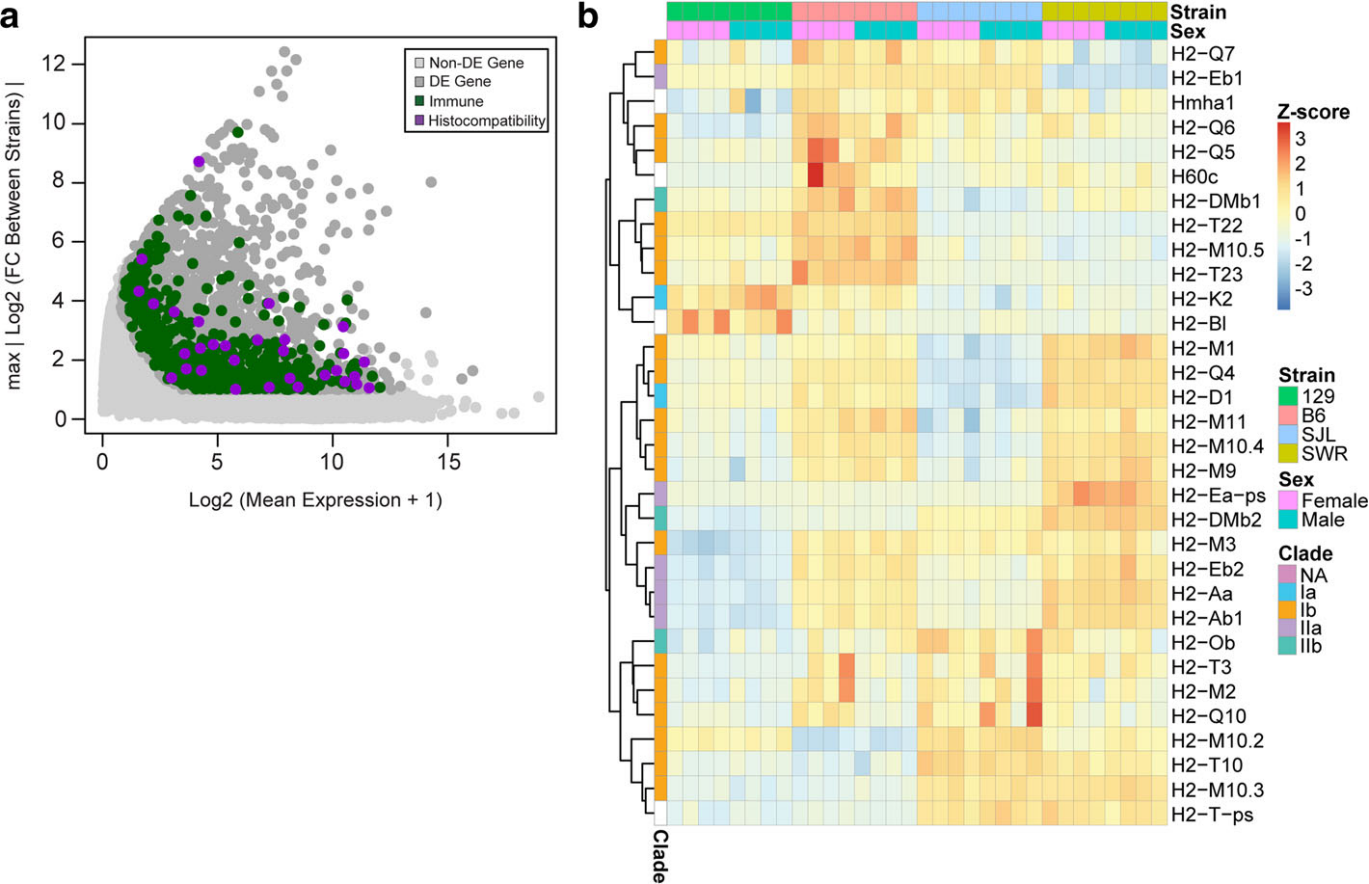
Differential expression of immune system related genes. **a** MA plot highlighting DE immune system related genes. Y-axis indicates the maximal value of FC between any two strains in log2 scale. Immune system genes are highlighted, including immune response gene (green) and MHC (purple). **b** Heatmap of the expression profile of the MHC genes in all four strains

An interesting observation was that 2159 immune system related genes were found to be expressed in VNO epithelia and 591 of them showed differential expression among strains (Fig. 5a). It was not clear whether these genes simply reflected the genetic background of the mice or contributed to the VNO mediated pheromone response. The largest group included 32 MHC genes, whereas others included five fragment receptor (Fce/g), eight guanylate binding protein (Gbp), five interferon induced (Ifit), 13 interleukin (Il), 11 interleukin receptors, and eight Toll-like receptor (Tlr) family genes (Fig. 5a). Interestingly, five of the immune system related genes were polymorphic pseudogenes with protein coding sequences known to be intact in other individuals of the same species.

The class I MHC molecules present peptide antigens derived from intracellular proteins to elicit immune responses. The expression of these genes was expected to be strain specific. Of the DE MHC genes, two were of class 1a, five were class IIa, and three were class IIb molecules (Fig. 5b). H2-Bl, a polymorphic pseudogene was also found to be DE. A subset of the MHC class 1b genes, specifically those of the H2-Mv family (*H2-M1, H2-M9*, *H2M10.2–5*, and *H2-M11*) have been shown to be co-expressed with specific clades of Vmn2r genes, namely *V2ra1–5* and *V2rc* [43–45]. They have been suggested to be either co-receptors of the Vmn2r products or to facilitate their expression on the VNO neuron surface.

### lncRNAs expression is correlated with chemoreceptors

**Fig. 6:**
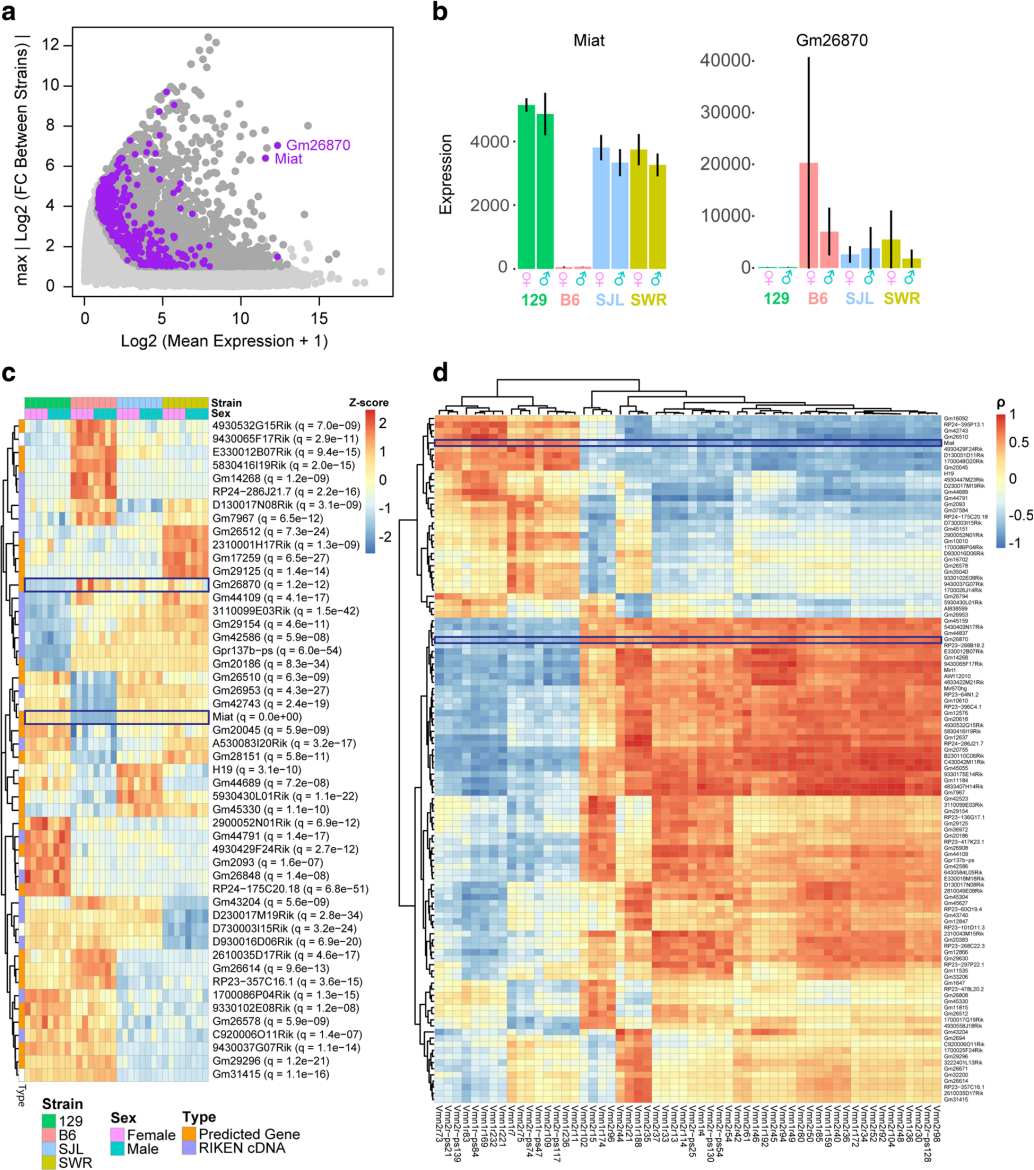
Differential expression of lncRNAs. **a** MA plot highlighting the 446 DE lncRNAs (purple). Y-axis indicates the maximal value of FC between any two strains in log2 scale. **b** Bar plot of mean normalized expression value of Miat and Gm26870 in all four strains. Error bars represent standard deviation of expression values. **c** Heatmap showing expression profiles of top 30 lncRNAs. The majority are gene model predictions and Riken transcripts. **d** Hierarchical analysis showing correlation between a subset of DE lncRNA and DE vomeronasal receptors that correlate highly with one another. Miat and Gm26870 are indicated

Long non-coding (lnc) RNA have emerged as major regulators of gene expression in cell differentiation and development [46–48]. We found 446 lncRNA biotypes from the DE gene set (Fig. 6a). The majority of these DE genes were gene models or Riken transcripts with unknown functions. Two highly expressed lncRNAs showed differential expression among the strains: *Gm26870* and *Miat* (Fig. 6b). Both genes showed exclusive expression profiles with high expression level in some strains and virtually undetectable in others (Fig. 6c). *Miat* was expressed highly by 129, SJL, and SWR strains, but at low levels in B6. *Gm26870* was expressed in B6 and Swiss Strain, but was virtually absent in the 129 strain with only a few samples in SWR showing very low expression.

We examined whether there was a correlation between the differentially-expressed lncRNAs and chemosensory receptor genes. Upon cluster analysis, we found that one group of lncRNAs, including *Miat*, was negatively correlated with a number of chemosensory receptor genes, and a second group was positively correlated with the rest (Fig. 6d). This finding implied a possible link between some of these lincRNAs and the differential expression of the chemosensory receptors.

### Strain and sex specific expression of genes

**Fig. 7:**
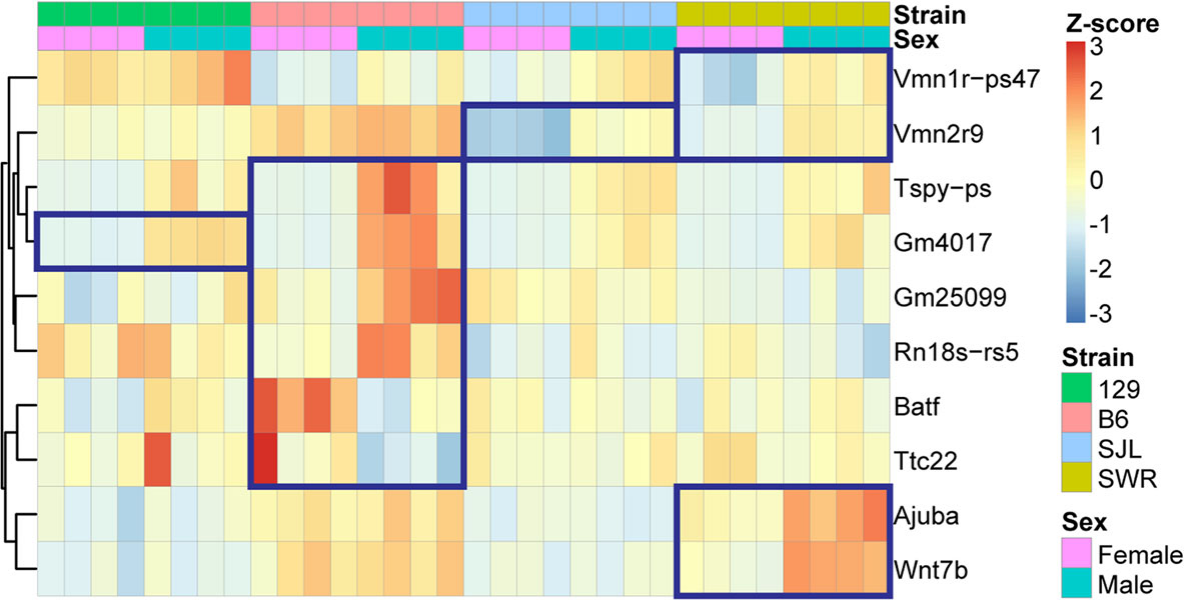
Differential gene expression between male and female animals within same strains. Heatmap showing expression profiles of ten genes exhibiting sex-specific expression within strains. Boxes indicate the strains within which significant sexual dimorphic expressions are found

Differentially expressed genes may be associated with specific combinations of sex and strain. These cases would be missed by our analyses when the data are aggregated in a phenomenon called the Simpson’s paradox [49]. Therefore, we performed an analysis to identify genes that showed DE between males and females within individual strains. We identified 10 genes that were differentially expressed in this specific manner: *Ajuba* (SWR)*, Vmn1r-ps47* (SJL and SWR), *Vmn2r9* and *Wnt7b* (SWR), 5 genes in B6 (*Batf, Gm4017, Gm25099, Rn18s-rs5* and *Ttc22) and Tspy-ps* (all 4 strains) (Fig. 7). Two of these genes, *Vmn2r9* and *Vmn1r-ps47,* encoded vomeronasal receptors and may mediate vomeronasal-based behaviors. No other gene has any known function in the VNO.

### Phylogenetic inference of strain lineage

**Fig. 8:**
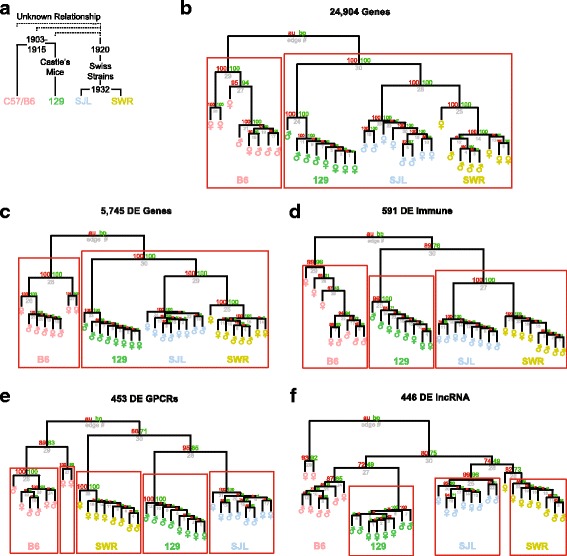
Phylogenetic analyses of DE genes. **a** The genealogy of strains. **b-f** Phylogenetic dendrograms of the strains derived from the gene expression profiles of all expressed genes (**b**), all DE genes (**c**), lncRNA (**d**), immune system related genes (**e**), and GPCRs (**f**). In all cases except in (**e** and **f**), B6 is an outgroup to all the other strains. In (**f**), 129 is no longer an outgroup to the Swiss strains. Abbreviations: au: approximately unbiased *p* value; bp: bootstrap probability

SJL and SWR strains originally diverged in 1920, with recombination occurring as late as 1932 [37]. The divergence between B6 and 129 mice occurred earlier, between 1903 and 1915 (Fig. 8a). Divergence of the strains can be reflected by nucleotide differences in the genes, as well as by differences in gene expression. Currently there is a lack of reference genomes that cover the strains we study here. Even though a rough reference genome exists for 129, close inspection of regions of the VR clusters indicate that they are thinly covered. In the absence of reference genomes, we built lineage relationships using gene expression level as traits and compared it to the breeding lineage map. Using genes with a normalized expression count above one, we generated a dendrogram of the strains. It revealed relationships among the strains that coincided well with the known lineage map, and suggested a closer relationship between 129 and the Swiss strains than with B6 (Fig. 8b, approximately unbiased *p* value au < 0.05). Similar phylogenetic relations were also established when all 5745 DE genes (Fig. 8c, au < 0.05), or 591 DE immune system related genes (Fig. 8d, au < 0.05) were used to generate the dendrograms. In contrast, using the 453 differentially expressed GPCRs, most of which are vomeronasal receptors, the phylogenetic relation no longer respected the pattern suggested by other gene groups. In this case, B6 is still an outgroup from the other strains (129, SJL, and SWR; au < 0.05), but 129 and SJL are closer to one another (au < 0.05) than the Swiss strains (Fig. 8e, au < 0.14). Interestingly, the tree from 446 DE long non-coding RNA transcripts (Fig. 8f, au < 0.05) also did not conform to the other gene sets.

## Discussion

Sensory neurons in the mammalian olfactory systems express the largest families of G-protein coupled receptors. Transcriptional regulation of these genes is highly coordinated to ensure each neuron expresses a unique set of genes. Through transcriptome analyses, we find that differentially expressed genes in the VNO are dominated by strain differences. A substantial number of GPCRs, as well as a chemosensory-related subclass of MHC family of genes, are differentially expressed among the strains. These genes are clustered in hotspot locations in the genome. A group of genes with unknown function, including many lncRNA genes and gene models, also show strain-specific expression. Intriguingly, our analyses reveal correlation and anti-correlation between lncRNAs and chemoreceptor genes, suggesting that they may be coordinately regulated. Importantly, we find that several chemoreceptors annotated as pseudogenes in the reference genome are expressed as functional genes due to SNPs in non-B6 strains. These discoveries hint at important differences of VNO functions in detecting pheromones and inter-species chemosensory cues.

### Sex specific gene expression

There is little evidence supporting sexually dimorphic gene expression in all strains. Except for *Xist* and Y chromosome genes, there are no other genes that can be considered as truly sexual dimorphic across strains. These X and Y chromosomal homologs have similar functions. *Eif2s3y* and *Ddx3y* are part of the translation initiation machinery [50]. Overexpression of *Eif2s3x* has been shown to substitute for loss of *Eif2s3y* [51] whereas the function of *Ddx3y* is thought to be replaceable by *Ddx3x* [52]. *Uty* is a putative histone demethylase, and *Utx* also a known histone demethylase [53]. *Kdm5d* and its X chromosome homolog *Kdm5c* (also known as *Jarid1d,c*) are both Lysine-specific demethylases and are functional homologs. The commonality between these genes pairs implies a dose-compensation mechanism that maintains the total product from X and Y chromosomes at constant levels in the VNO. When expressions from both sex chromosomes are considered, the overall expression levels of these genes no longer have significant difference.

We do not find any GPCRs, proteins associated with pheromone binding such as lipocalins or MHC proteins, as differentially expressed between the sexes. This observation is consistent with previous work suggesting no significant sexual dimorphism in the expression of receptors recognizing sex pheromones or in VNO response to urine stimulation [16, 39]. The absence of significant receptor differences suggests that sexually dimorphic behavioral responses are unlikely to originate from the VNO. They are more likely to be the result from the differential processing of pheromone cues in the brain circuitry [54].

Previous studies have implicated a few genes to be differentially expressed between the sexes [15]. While our study replicates these results, our data show that these differences are restricted to the strains examined. Products encoded by these genes may still contribute to differential function of the VNO in the B6 strain, but the differences cannot be generalized.

### Strain-related differential gene expression

With respect to strains, the largest group of DE genes is related to immunological functions, including genes encoding MHC, cytokines and their receptors, as well as molecules involved in signaling pathways. It is well established that inbred strains of mice are distinguished by their haplotypes and all cells express MHC molecules. Therefore, it is not surprising that the MHC genes in the VNO also show strain differences as in other tissues. The differential expression of H2-Mv non-classic MHC molecules, on the other hand, may have implications in chemosensation. These genes have been shown to be coordinately expressed with V2r subfamilies V2ra1–5 and V2rc [43–45] and have been shown to allow for ultra-sensitive pheromone detection, possibly by influencing V2r surface expression [55]. Differential expression in the H2-Mv genes may affect the affinity and sensitivity of V2rs to specific ligands, and their recognition by the animals. In this context, these DE H2-Mv genes could affect strain-specific recognition.

### Strain-specific expression of chemosensory receptors

Despite the relatively recent lineage separation of different inbred lab strains, we find abundant examples of DE genes in the VNO. These differences include binary expression differences and modulated expression levels. In striking examples, we find SNPs that render some of the annotated pseudogenes functional in some strains, including both V1rs and V2rs.

The expression of particular sets of receptors may define the cue set each strain of mice can detect. Different clades of receptors appear to be tuned to specific sets of cues. We found these receptors exhibit differential expression among the strains. The expression of *Vmn1r85* is high in only B6 mice while *Vmn1r185* is high in all strains except 129 mice. Notably, the SJL is considered a ‘challenging breeder’ by Jackson Laboratories. It is possible that a reduced response to estrus cues may cause reduced mating in this strain.

Besides the V1rj and V1re clades, differentially expressed V1rs are found in all other clades except L, including eight members of V1ra, six of V1rb, and 17 of V1rc. Deletion of a genomic region encompassing both V1ra and V1rb genes results in decline in mating and aggression [56]. The V1rc receptors have been implicated in detecting cues present in female mice or predators. Differential expression of these V1rs may affect the recognition of environmental as well as species-specific cues.

The V2rs have long N-terminus domains and have been shown to recognize polypeptide pheromones. V2r-expressing cells respond robustly to MHC peptides and can also be activated by the MUPs [57, 58]. As these polypeptides may be specifically associated with strain and individuals, differentially expressed V2rs may lead to divergent recognition of strain information and trigger biased responses.

The expression of *Fpr-3* shows strain differences. Formyl peptides are present in the mitochondria of bacteria and are released when bacteria die. The presence of formyl peptides triggers chemotaxis of immune cells in response to infection. FPR expression in the VNO is thought to allow the animals to detect the health status of other animals [9]. The differential expression *Fpr-3* may bias this recognition.

Taken together, differential expression of the VRs may lead to the recognition of a particular set of cues in one strain but not the other. It is worth noting, however, that even though the VRs are highly specific in their ligand recognition, there is certain redundancy in how pheromones are recognized. For example, *Vmn1r85* (*V1rj3*) and *Vmn1r89* (*V1rj2*) receptors are activated by sulfated estrogens, but they display different sensitivities to the ligands [39, 59]. Given that many of the differentially expressed VRs have paralogs in the genome, differential expression of the VRs may reduce or enhance the sensitivity to certain pheromones, rather than create a situation in which a pheromone is recognized by one strain but not the other.

### lncRNAs

Both genetic and epigenetic mechanisms may contribute to differential gene expression among the strains. We find strong anti-correlation between the expression of *Miat* and *Gm26870*, two lncRNAs, which along with others show strong positive and negative correlations with chemoreceptor expression. lncRNAs are expressed highly in the nervous system [60] and are known to control gene expression by directly regulating gene-specific transcription and splicing, as well as epigenetic modifications [46, 61]. *Miat*, also known as *RNCR2* or *Gamufu* [62, 63], is one of the most strongly DE lncRNAs among the strains. It is known to regulate cell specification in the developing retina [62]. While the functional roles played by *Miat* and the other lncRNAs in regulating VNO gene expression are not clear, the strong correlations among the transcripts raised the possibility that they may be coordinately regulating differential gene expression among the strains.

### Implication in strain evolution

Although differences in gene expression are not equivalent to genetic differences at the nucleotide level, they are nonetheless important traits that can provide information about evolutionary divergence among the mouse strains. The phylogenetic relationship inferred from the expression of GPCR genes does not conform to those by other genes, nor to that of the genealogy. Several VNO receptor genes marked as pseudogenes in the reference B6 genome are functional in other strains. Moreover, we find SNPs that result in synonymous and missense changes in protein coding in many V1r and V2r genes. These observations, together with the observation that several hotspots of DE genes are enriched in VNO receptors, suggest that the VNO receptors genes and their expression may have followed a different evolutionary path from the rest of the genome. These differentially expressed chemosensory receptors may enable different strains of mice to sense social cues emitted by conspecific animals, react to the health status of another animals, or respond to heterospecific signals including predators in distinct manners. The differential detection of social cues may therefore underlie some of the strain-specific behavior differences observed in mice.

## Conclusions

Transcriptome analyses provide little support of sexual dimorphism in gene expression in the VNO. In contrast, there are profound variations in the expression of immune response related genes, vomeronasal and G-protein coupled receptor genes among different strains of mice. These differentially expressed genes are concentrated in hotspots on the genome, indicating rapid evolution of genes involved in pheromone detection. These findings suggest it is likely that diverse strains of mouse perceive pheromone cues differently. Behavioral difference among strains in response to pheromone may thus first arise from differential detection of pheromones by the vomeronasal organ. On the other hand, sexually dimorphic responses to pheromones more likely originate from dimorphic neural circuits in the brain than from differential detection.

## Methods

### RNA library preparation & sequencing

All strains of animals were purchased from Jackson laboratory. Mice are maintained in Lab Animal Services Facility at Stowers Institute with a 14:10 light cycle, and provided with food and water ad libitum. Experimental protocols were approved by the Institutional Animal Care and Use Committee at Stowers Institute and in compliance with the NIH Guide for Care and Use of Animals. Total RNA was isolated from VNO epithelia of individual mouse using TRIzol solution (Thermo Fisher Scientific) followed by spin-column (Zymo Research) purification. Ribodepletion was performed using Ribo-Zero Gold rRNA Removal kit (Illumina) to remove rRNA from the sample prior to library preparation. Sequencing libraries were generated using TruSeq Stranded Total RNA Kit (Illumina) and sequenced as 125 bp paired-end stranded reads on Illumina Hi-Seq 2500 platform. Preliminary analysis including basecalling was performed using HiSeq Control Software (v2.2.58) with fastq files generated using bcl2fastq. FastQC [64] reports were generated for each sample to ensure sequencing quality. Trim Galore was used with default parameters to trim reads with leftover adapter sequence and low quality scores [65].

### Sequence alignment

GRCm38 (mm10) mouse reference genome was used to align the reads with STAR aligner version 2.5.2b (Dobin, et al. 2013). Ensembl reference annotation version 87 [66] was used to define gene models for mapping quantification. Uniquely mapped reads for each gene model were produced using STAR parameter “--quantMode GeneCounts” and raw stranded counts were extracted from the fourth column of the output matching the orientation produced by the True-seq stranded preparation protocol used. All the options chosen are equivalent to the HTSeq command “htseq-count option -s reverse”.

### Differential expression analyses

Differential Expression analysis was performed using the R package DESeq2 [67]. Under the assumption of negative binomial distribution, we normalized the data for technical variation in sequencing depth among each sample. Each gene was then fit to a generalized linear model and dispersion coefficients were tested using cooks distance for independent filtering of high variance genes. For genes that passed independent filtering, Log_2_ fold changes (LFC) between groups and their standard errors were used in a Wald test for differential expression. Genes were considered differentially expressed if any of groups passed independent filtering and had a FC > 2 and FDR < 0.05.

### Additional downstream analyses

For PCA analysis, we used DESeq2 internal methods to calculate and plot principal components using all expressed genes instead of the default top 500 varying genes. Gene expression heatmaps were created with the R package pheatmap using regularized log transformed normalized counts from DESeq2 [68]. GO analyses on the groups of DE genes were performed in R using topGO [69] and based on GO annotations from BiomaRt [70]. To identify hot spots that contained a high percentage of DE genes, we used rollapply from the zoo package (https://cran.r-project.org/web/packages/zoo/index.html) to create sliding windows of 25 expressed genes and slid the window across each chromosome separately to calculate the probability of observing DE genes that exceed random chance. Within the sliding window, we performed the Poisson test using the function ppois to compare the percentage of DE genes within the window with the percentage of DE genes in the entire genome. Data were visualized using GenomicRanges [71] and ggbio [72].

Tracks for SNP identification and visualization were created using Integrative Genomics Viewer [73]. Identified SNPs were incorporated into the reference sequences from Ensembl, and translated to proteins using the ExPASY online translate tool [74], aligned using ClustalW [75], and visualized using MView [76] through the EMBL-EBI online web services [77]. Homologous sequences were identified using NCBI’s Blastn and Blastp [78]. Correlation analysis dendrograms were created in R by running PVclust [79] using the ‘average’ method for clustering and a custom spearman implementation for calculating distance, parallelized with 10,000 bootstraps.

## Additional files

Additional file 1: Figure S1. Distribution of uniquely mapped, multi-mapped and unmapped reads among the samples presented as total reads (A) and percentage of reads (B). (PDF 464 kb)

Additional file 2: Figure S2. Differentially expressed chemosensory genes other than V1r and V2r families. (PDF 138 kb)

Additional file 3: Figure S3. Strain-specific expression of vomeronasal receptors. Data is displayed as expressed (blue) or not expressed (white) to highlight the exclusive patterns of expression for some of the genes. (PDF 87 kb)

Additional file 4: Figure S4. High degree of polymorphism and differential expression of VR genes among strains. (**A-F**) Example track files illustrating the mapping of reads to individual VR genes. Each track is a superposition of four individual samples with SNPs highlighted as vertical lines with substitutions represented as follows: thymine as red, guanine as brown, cytosine as blue, and adenine as green. (PDF 4541 kb)

Additional file 5: Figure S5. Differences in expression level among different clades of VRs. (**A**) Expression of all V1r clades are represented in all strains. Clade J receptor genes are more highly expressed than receptors of other clades. (**B**) Expression of all V2r clades are represented in all strains. (PDF 558 kb)

Additional file 6: Figure S6. Polymorphism and differential expression of FPR genes. A) Fpr-rs3. B) Fpr3. (PDF 2426 kb)

Additional file 7: Figure S7. Sequence comparison of functionalized pseudogene. Alignment of *Vmn1r-ps27* from SWR with *Vmn1r-ps27* and Vmn1r42 from B6. (PDF 814 kb)

## Abbreviations

DE: Differentially expressed
FC: Fold change
FDR: False discovery rate
FPR: Formyl peptide receptor
GO: Gene ontology
GPCR: G-protein coupled receptor
LFC: Log_2_ fold change
MHC: Major histocompatibility complex
ORF: Open reading frame
PC: Principal component
PCA: Principal component analysis
VNO: Vomeronasal organ
VR: Vomeronasal receptor
