# Supplementary figures and images for: Pronounced strain-specific chemosensory receptor gene expression in the mouse vomeronasal organ
Source: BMC Genomics. 2017 Dec 12;18:965. doi: 10.1186/s12864-017-4364-4 (PMC5727874; doi:10.1186/s12864-017-4364-4)

**A**

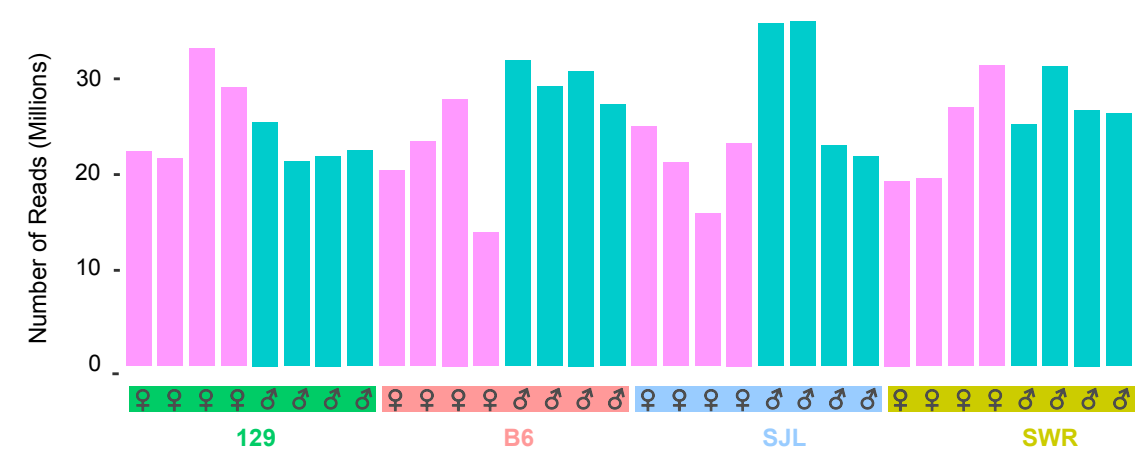

**B**

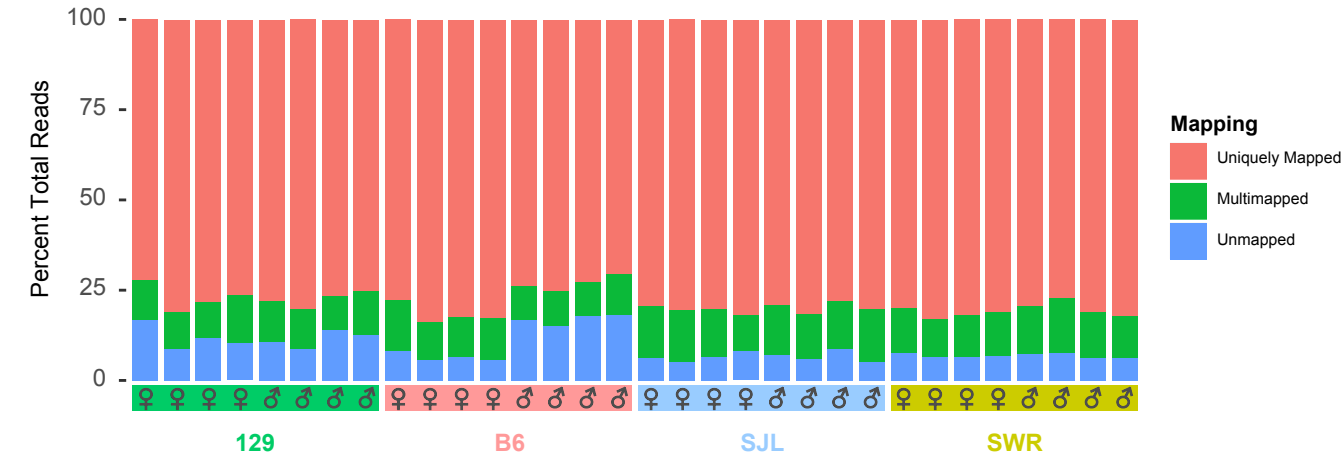

Figure S1

Supplement: Supplementary file 1 — Distribution of uniquely mapped, multi-mapped and unmapped reads among the samples presented as total reads (A) and percentage of reads (B). (PDF 464 kb) [file 12864_2017_4364_MOESM1_ESM.pdf]

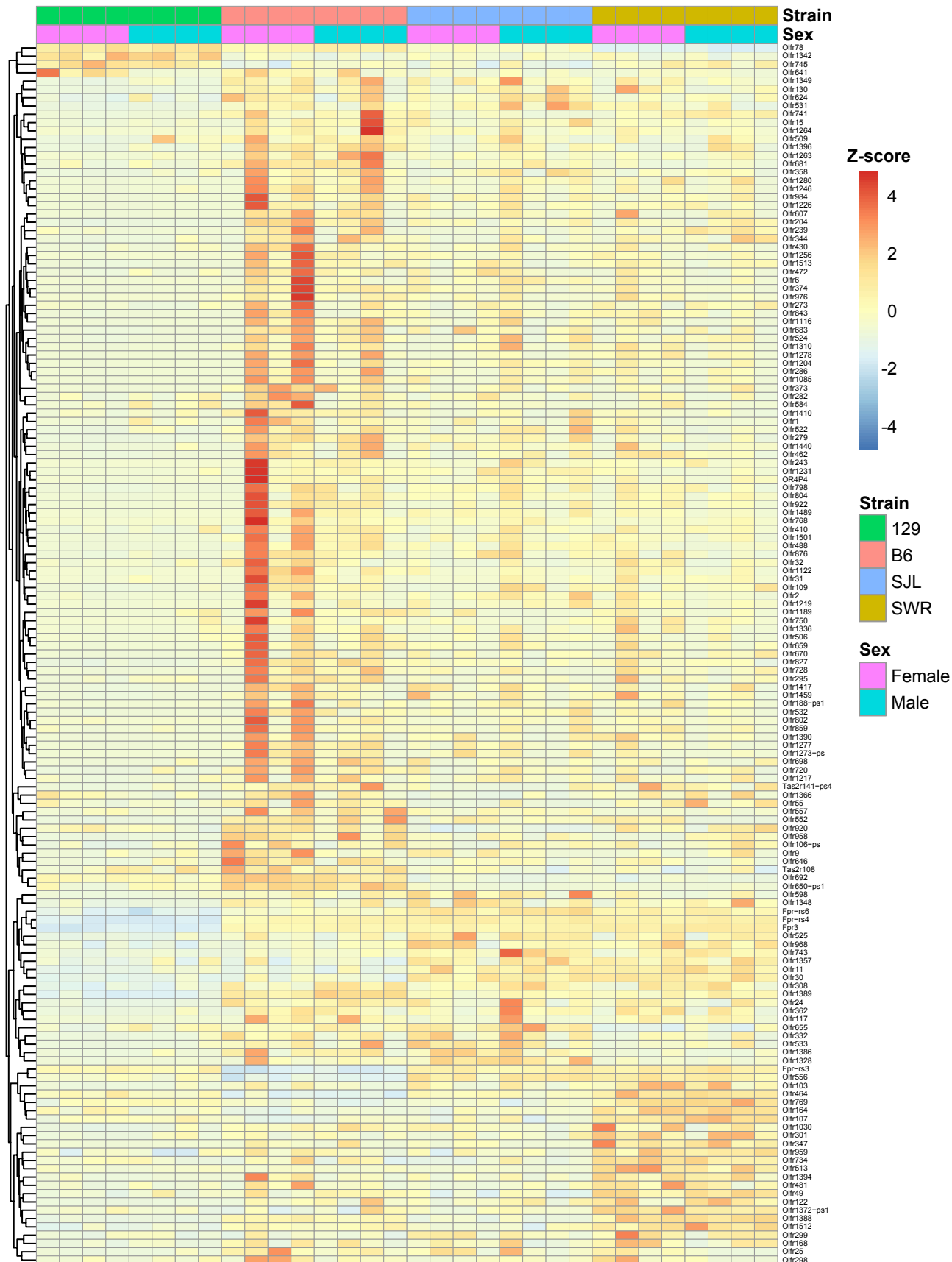

Figure S2

Supplement: Supplementary file 2 — Differentially expressed chemosensory genes other than V1r and V2r families. (PDF 138 kb) [file 12864_2017_4364_MOESM2_ESM.pdf]

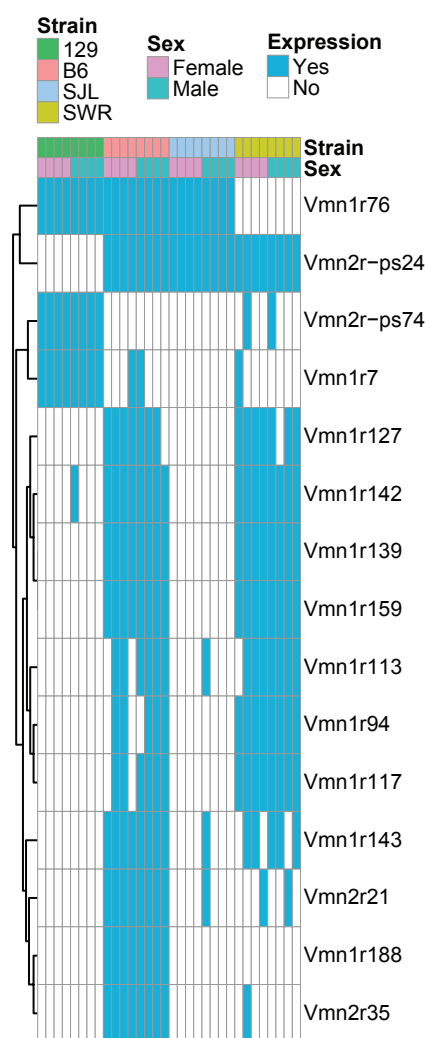

Figure S3

Supplement: Supplementary file 3 — Strain-specific expression of vomeronasal receptors. Data is displayed as expressed (blue) or not expressed (white) to highlight the exclusive patterns of expression for some of the genes. (PDF 87 kb) [file 12864_2017_4364_MOESM3_ESM.pdf]

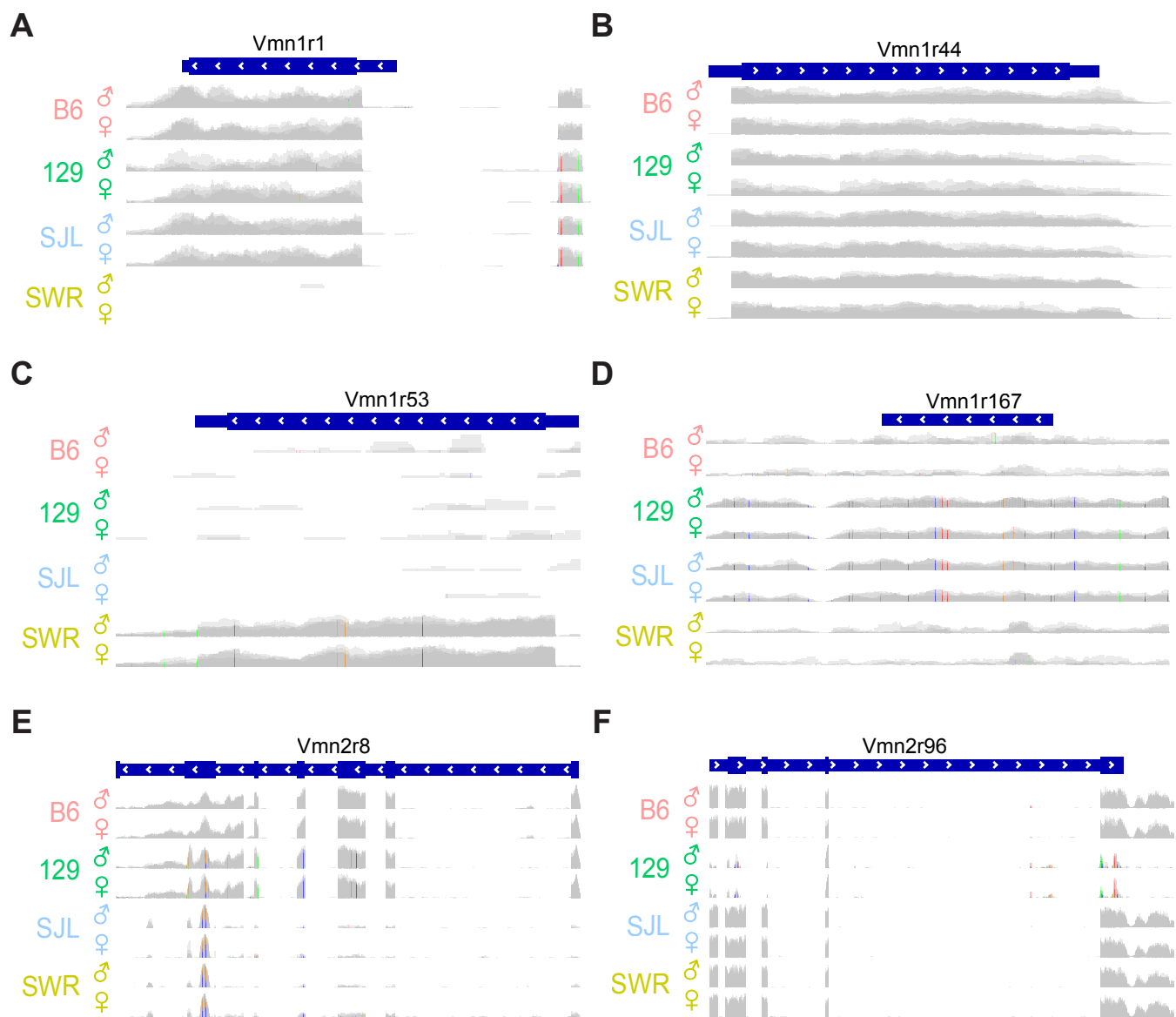

Figure S4

Supplement: Supplementary file 4 — High degree of polymorphism and differential expression of VR genes among strains. (A-F) Example track files illustrating the mapping of reads to individual VR genes. Each track is a superposition of four individual samples with SNPs highlighted as vertical lines with substitutions represented as follows: thymine as red, guanine as brown, cytosine as blue, and adenine as green. (PDF 4541 kb) [file 12864_2017_4364_MOESM4_ESM.pdf]

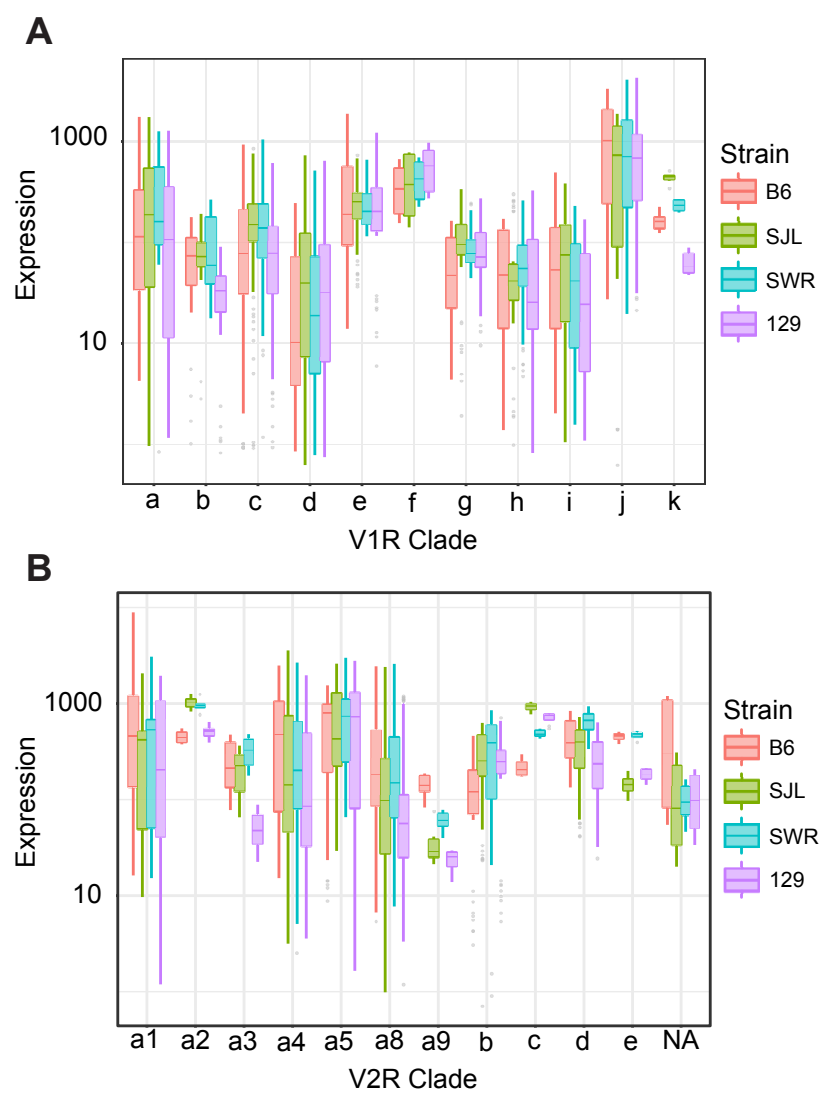

Figure S5

Supplement: Supplementary file 5 — Differences in expression level among different clades of VRs. (A) Expression of all V1r clades are represented in all strains. Clade J receptor genes are more highly expressed than receptors of other clades. (B) Expression of all V2r clades are represented in all strains. (PDF 558 kb) [file 12864_2017_4364_MOESM5_ESM.pdf]

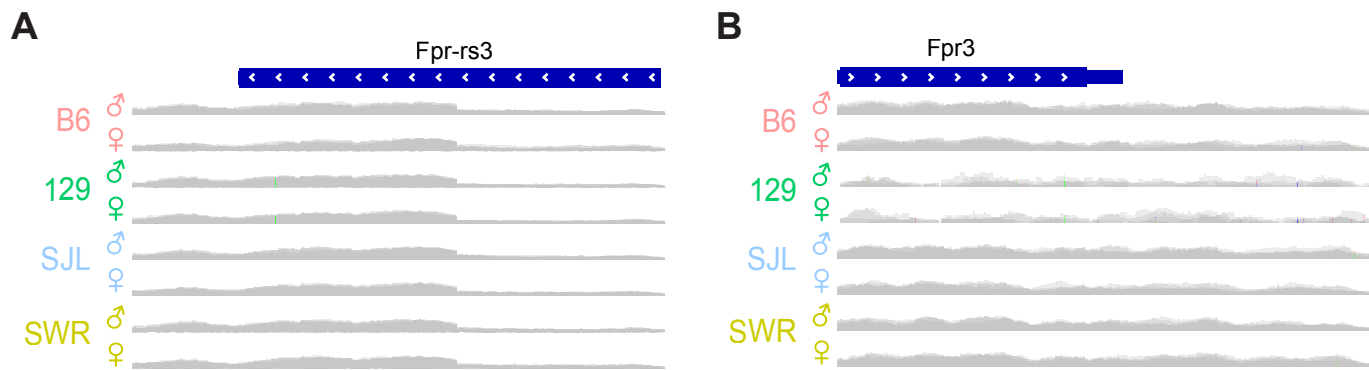

Figure S6

Supplement: Supplementary file 6 — Polymorphism and differential expression of FPR genes. A) Fpr-rs3. B) Fpr3. (PDF 2426 kb) [file 12864_2017_4364_MOESM6_ESM.pdf]
