# Supplementary material for: Pronounced strain-specific chemosensory receptor gene expression in the mouse vomeronasal organ
Source: BMC Genomics. 2017 Dec 12;18:965. doi: 10.1186/s12864-017-4364-4 (PMC5727874; doi:10.1186/s12864-017-4364-4)
Supplement: Supplementary file 7 — Sequence comparison of functionalized pseudogene. Alignment of Vmn1r-ps27 from SWR with Vmn1r-ps27 and Vmn1r42 from B6. (PDF 814 kb) [file 12864_2017_4364_MOESM7_ESM.pdf]

```
Vmn1r-ps27 (B6)  ATGAGTGAGATTCTGTTCTTTTCTCCTCAGCCACTGTTCCCATGTACAATGAATAAGAACAGTAGACTACATACTAATTCTAACATAGGGAACATATTTTTCTCCGAAATTGGCATTGGC  120
Vmn1r-ps27 (SWR) ATGAGTGAGATTCTGTTCTTTTCTCCTCAGCCACTGTTCCCATGTACAATGAATAAGAACAGGAGACTACATACTAATTCTAACATAGGGAACATATTTTTCTCAGAAATTGGCATTGGC  120
Vmn1r42 (B6)     ATGGGTGATATTCTGTTCTCTTCTCCTCAGTCAATGTTCTCACATACAATGAATAAGAACAGCATACTACATACTCATTCTATCATAGGAAAAACATTTTTCTCTGAAATTGGCATTGGG  120

Vmn1r-ps27 (B6)  ATCTCAGGAAACAGCTTCCTTCTTCTCTTCCAAATCCTCAAGTTCATTCGTGGGCACAGGCCCAGACTCACTGACCTGCCCATTGGTCTCTTGTCCCTTATCCACCTACTGATGCTACTG  240
Vmn1r-ps27 (SWR) ATCTCAGGAAACAGCTTCCTTCTTCTCTTCCAAATCCTCAAGTTCATTCGTGGGCACAGGCCCAGACTCACTGACCTGCCCATTGGTCTCTTGTCCCTTATCCACCTACTGATGCTACTG  240
Vmn1r42 (B6)     ATCTCAGGCAACAGCTTCCTTCTTCTTGTCCACATCCTAAAGTTCATTCGTGGGCACAGGCCCAGACTCACTGACCTGCCCATTGGTCTCTTGTCCCTAATCCACCTACTGATGCTACTG  240

Vmn1r-ps27 (B6)  GTCATGGCATTCATAGCCATAGACATTTTTATTTCTTGGAGGGAATGGGATGACATCATACGTAAATTCCTTCTGTACCTGTACAG..TTTTGAGGGGTTTCTCCCTTTGCACCACCAGC  358
Vmn1r-ps27 (SWR) GTCATGGCATTCATAGCCATAGACATTTTTATTTCTTGGAGGGAATGGGATGACATCATAAGTAAATTCCTTCTGTACCTGTACAGAGTTTTGAGGGGTTTCTCCCTTTGCACCACCAGC  360
Vmn1r42 (B6)     GTCGCAGCATTCATAGCCACAGACATTTTTATCTCTCGGAGGGGCTGGGATGACATCATATGTAAATTCCTTGTGTACCTGTACAGAGTTTTGAGGGGTTTCTCCCTTTGCACCACCAGC  360

Vmn1r-ps27 (B6)  ATGTTGAGTGTCCTCCAGGCTATCATACTTAGTCCCAGAAGCTCCTGTTTAGCAAAGTTCAAGCATAAATCTCCTCATCACATCTCATGTACCATTATTTTCCTGAGTGTCCTCTATATG  478
Vmn1r-ps27 (SWR) ATGTTGAGTGTCCTCCAGGCTATCATACTTAGTCCCAGAAGCTCCTGTTTAGCAAAGTTCAGGCATAAATCTCCTCATCACATCTCATGTACCATTATTTTCCTGAGTGTCCTCTATATG  480
Vmn1r42 (B6)     ATGTTGAGTATCCTCCAGGCCATCATTCTTAGTCCCAGAAGCTCCTGTTTAGCAAAGTTCAAGCATATATCTCCTCATCACATATCAGGTGCCATTCTTTTCCTGAGTGTCCTCTATATG  480

Vmn1r-ps27 (B6)  TTAATTGGCAGTCACCTCTTG...TCCATCATTGCTACCCCGAATTTGACCACAAATGACTTCATTTATATTACTCAATCCTGCTCCATTCTATCCTTGAGTTACCTCATGCAAAGCATA  595
Vmn1r-ps27 (SWR) TTAATTGGCAGTCACCTCTTG...TCCATCATTGCTACCCCGAATTTGACCACAAATGACTTCATTTATATTACTCAATCCTGCTCAATTCTATCCTTGAGTTACCTCATGCAAAGCATA  597
Vmn1r42 (B6)     TTAATTGGCAGTCAACTCTTGGTATCCATCATTGCCACCCCCAATTTGACCATGAATGACTTTATTTATGTTACTCAGTCCTGCTCTATTCTACCCTTGAGTTACCTCATGCAAAGCATA  600

Vmn1r-ps27 (B6)  TATTCTACGCTGGTAGCCATCAGAGAAGTTTTTCTTATTAGTCTCATGGTCCTCTCTAATTGGTACATGGTGGCCCTCTTGTGCATGTACAGGAAACGGACCCAGCATCTTCAACGATCC  715
Vmn1r-ps27 (SWR) TATTCTACGCTGGTAGCCATCAGAGAAGTTTTTCTTATTAGTCTCATGGTCCTCTCTAATTGGTACATGGTGGCCCTCTTGTGCATGTACAGGAAACTGACCCAGCATCTTCAACTATCC  717
Vmn1r42 (B6)     TATTCTACACTGCTAGCCATCAGGGAATTCTTTCTTATTAGTCTCATGGTCCTCTCTAATTGGTACATGGTAGCCCTCTTAAGCATGCACAGGAAACAGACACAGCATCTTCATGGAACC  720

Vmn1r-ps27 (B6)  ATACTTTCCCAAAAAAAAATCCCCAGAGCAAAGTGCCACCCAGACCATCCTGATGCTCATGAGCTTCTTTGTGCTGATGACCATCTATGACATCATGGTCTGCTGCTCAAGAGCTATGTTC  835
Vmn1r-ps27 (SWR) ACACTTTCCCAAAAAAAAATCCCCAGAGCAAAGTGCCACCCAGACCATCCTGATGCTCATGAGCTTCTTTGTGCTGATGACCATCTATGACATCATGGTCTGCTGCTCAAGAGCTATGTTC  837
Vmn1r42 (B6)     AACCTGTCCCCCAAAAAATCCCCAGAGCAAAGTGCCACCCAGACCATCCTGATGCTCATAAGCTTCTTTTTGCTGATGACCATCTATGACACCATAGTCTCCTGCTCAAGAACTATGTTC  840

Vmn1r-ps27 (B6)  CTGAATGATCCAACAACTTACTCTATCAAACTCTTTATAATGCACATTTATGCCACTGTCAGCCCTTTTGTGTTTATGAGCACTGAAAAACATATAGTTAACTTTTTGAGATCCATGGGT  955
Vmn1r-ps27 (SWR) CTGAATGATCCAACAACTTACTCTATCCAACTCTTTATAATTCACATTTATGCCACTGTCAGCCCTTTTGTGTTTATGAGCACTGAAAAACATATAGTTAACTTTTTGAGATCCATGGGT  957
Vmn1r42 (B6)     CTGAATGATCCAACATCTTACTCTATCGAACTCTTTATAATGCACATCTATGCCACTGTCAGCCCTTTTGTGTTTATGAGCACTGAAAAACATATAGTTAACTTTTTGAGATCCTTGGGT  960

Vmn1r-ps27 (B6)  AAGAGGGTGATAAATTTTAATCTTCACTGA  985
Vmn1r-ps27 (SWR) AAGAGGGTGATAAATTTTAATCTTCACTGA  987
Vmn1r42 (B6)     AAGAGGGTGATAAATTTTAATCTTCATTGA  990
```

Figure S7
